# Bridging Operative Standards to Clinical Practice: A Case Comparison of Synoptic Operative Report Implementation in Breast Cancer Surgery

**DOI:** 10.1245/s10434-025-19042-6

**Published:** 2026-01-27

**Authors:** Meagan Elam, Jamie Hillas, Rachel Moyal-Smith, Tasleem J. Padamsee, Sarah A. Birken, Mary Brindle, Ko Un Park

**Affiliations:** 1 Ariadne Labs, Brigham and Women’s Hospital, Harvard T.H. Chan School of Public Health, Boston, MA USA; 2 Division of Breast Surgery, Department of Surgery, Brigham and Women’s Hospital, Boston, MA USA; 3 AstraZeneca, Wilmington, DE USA; 4 James Comprehensive Cancer Center, The Ohio State University, Columbus, OH USA; 5 Wake Forest University School of Medicine, Winston-Salem, NC USA; 6 Department of Surgery, Alberta Children’s Hospital, Cumming School of Medicine, University of Calgary, Calgary, Canada; 7 Breast Oncology Program, Dana-Farber Brigham Cancer Center, Boston, MA USA; 8 Harvard Medical School, Boston, MA USA

**Keywords:** Commission on cancer, Synoptic operative report, Implementation science, Breast cancer surgery, Consolidated framework for implementation research, Accreditation standard

## Abstract

**Background:**

In 2020, the Commission on Cancer (CoC) announced a new accreditation standard requiring use of synoptic operative reporting (SOR) to improve the proportion of patients with breast cancer receiving guideline concordant care. We conducted a case-comparison of four CoC accredited sites’ implementation of breast SOR to identify ways in which the CoC could address common implementation challenges.

**Methods:**

We conducted semi-structured interviews from December 2021 to May 2022 with 31 stakeholders purposefully sampled from two NCI-Designated Cancer Programs and two Comprehensive Community Cancer Programs in the US. We used the Consolidated Framework for Implementation Research to guide thematic analysis and identify similarities and differences between cases.

**Results:**

All cases expressed that the CoC was the driver for SOR implementation, had concerns about complexity and costs, and noted that the cancer liaison physician and information technology are critical during implementation. Sites noted that surgeons who dictate had challenges changing workflows to accommodate SORs. All sites believed standardization and data collection via SORs were beneficial but not essential, leading to low tension for change. Informatics support and clinician expertise with information technology varied by site, affecting the ability to translate clinical requirements for SOR integration.

**Conclusions:**

Cancer liaison physicians were a critical champion across all settings, underscoring the importance of the CoC’s continued engagement with this role. To reduce cost and complexity of developing SORs and account for varying clinical informatics knowledge and resources, the CoC should consider providing additional tailored technical support.

**Supplementary Information:**

The online version contains supplementary material available at 10.1245/s10434-025-19042-6.

Adherence to the evidence-based guidelines for surgical care of breast cancer has been shown to improve patient outcomes. However, rates of adherence to the critical elements of a breast cancer operation (e.g., negative margin status, adequate lymph node retrieval) are challenging to monitor through current documentation, which largely consists of long-form, narrative operative notes. A recent analysis of data from the National Cancer Database found that up to one-third of patients with breast cancer did not receive standard-concordant care, and those who did had a longer median overall survival.^1^

Synoptic operative reporting (SOR) has been found to improve standardization of care and adherence to best practices in treatment and reporting guidelines in cancer care.^2–5^ Synoptic operative reporting are electronic, templated checklist-based reports that record key technical aspects of the surgery in a standardized format. In contrast to narrative operative notes, SORs facilitate identification of critical information needed for adjuvant treatment decisions for other members of the care team and abstraction of information for quality metrics and research.^2–4,6–8^

In 2020, the Commission on Cancer (CoC), an accreditation program that includes approximately 1,400 accredited cancer centers accounting for 70% of cancer care in the United States, announced a new accreditation standard requiring SOR use to document specific technical elements of lymph node retrieval procedures as part of the full operative note for breast cancer surgeries.^9^ Examples of these technical elements to be documented include the use of tracers to identify sentinel lymph nodes (CoC accreditation standard 5.3) and removal of Level I and II lymph nodes with preservation of the main nerves of the axilla for axillary dissection procedures (CoC accreditation standard 5.4). To support the implementation of SORs, the CoC released implementation guidance that focused on surgeon education.^10^ Synoptic operative reporting education may be of limited benefit because of features of surgeons’ practice contexts that influence implementation success.^11–13^ This especially holds true because we previously identified implementation barriers for SORs cannot be addressed through education resources alone (Alliance A20_Pilot9).^9^ Recent studies have demonstrated that noncompliance with these operative standards were initially low without further intervention.^14,15^ Studying the implementation of CoC standards is critical since adoption of previous CoC standards (e.g., psychosocial distress screening, survivorship care plan) varied widely across sites due, in part, to insufficient implementation resources.^16,17^ The goal of this case-comparison study of four sites (two academic centers and two community hospitals) is to identify additional implementation resources that could be tested to determine their impact on improving adoption of the SOR accreditation standards. Understanding similarities in context, knowledge and attitudes, and implementation processes is likely to provide insights on additional implementation support and resources that address common needs and may improve uptake of standards on a larger scale, while understanding differences in context may reveal areas of implementation that require more tailoring to fit a site’s needs.

## Methods

### Study Design

This was a planned secondary analysis of our previous Alliance A20_Pilot9 study, wherein we reported the composite analysis of barriers to implementing breast SORs across four different CoC-accredited institutions in the United States: two NCI-Designated Cancer Programs and two Comprehensive Community Cancer Programs.^9^ In this study, we conducted a qualitative cross-case comparison study of these four CoC-accredited institutions to better understand how SOR implementation was affected by different combinations of contextual factors at each of the sites.^18^

### Conceptual Frameworks

The Consolidated Framework for Implementation Research (CFIR) is an implementation science determinant framework with five domains (outer setting, inner setting, individual characteristics, intervention characteristics, process) and multiple constructs and subconstructs that are associated with implementation success. The Theoretical Domains Framework (TDF) is another determinant framework designed for implementation research that specifically focuses on identifying influences on individual health professional behavior as it relates to implementation of evidence-based practices. The TDF is a combination of 33 theories of behavior and behavior change organized into 14 domains.^19–21^

### Study Sample and Data Collection

Details of qualitative data collection methods have been reported previously.^9^ Briefly, we conducted semistructured, in-depth interviews across the four study sites with multidisciplinary healthcare professionals with roles in breast SOR implementation, as described previously.^9^ Study sites were purposively selected to reflect varied institution types, electronic medical record (EMR) vendors, and geographic regions of the country. We combined the CFIR and TDF frameworks to create the interview guide (supplemental file).^22^ The codebook for the interview based on CFIR and TDF domains was previously published.^9^ We used CFIR constructs to assess external and organizational contextual factors, whereas TDF was used to assess characteristics of the healthcare professionals. Interviewees included breast surgeons, cancer program administrators, and information technology (IT) leaders and analysts. Informed consent was obtained from all participants. All interviews were conducted between December 2021 and May 2022, audio recorded, and transcribed verbatim.

### Data Analysis

For the cross-case comparison, we defined each site as a case and aimed to assess for similarities and differences associated with each of the CFIR constructs. Because we were focusing more on similarities and differences at the case level, we mapped all TDF codes used to assess individual-level factors to the CFIR Characteristics of Individuals constructs using the mapping outlined in O’Donovan et al.^23^ Within each of the five CFIR domains, all interviews were previously deductively coded to the applicable construct or subconstruct. To ensure reliability in coding for each transcript, the two coders previously independently coded a portion of each transcript, came together to discuss and clarify codes, and resolved any discrepancies. Agreement between coders was measured using the Cohen’s kappa coefficient following initial coding.^9^

For this analysis, we created a matrix on a spreadsheet with each case as a column and CFIR constructs and subconstructs as rows. Two qualitative analysts (ME and JH) with guidance (KP) conducted a secondary thematic analysis. Code summaries that contained excerpts from all interviews were summarized for each case. The similarities and differences in context, knowledge and attitudes related to SORs, and implementation strategies for each case were synthesized into themes for each CFIR construct. The qualitative analysts met periodically to ensure consistency in construct definition interpretation and alignment on the identified themes. Analysts then tabulated the number of cases with similarities in the themes expressed through their interviews within each of the constructs and subconstructs. To align with the goals of qualitative research to capture the range and nuance of perspectives within a case, themes were counted if they were discussed by at least one interviewee at a case. Similar to methods used by Schmitz and Finkelstein, themes common across the four cases were counted and a heatmap was generated to aid in pattern recognition.^24^

We reported data according to the Consolidated criteria for reporting qualitative research (COREQ) guidelines.^25^ This study was approved by Dana-Farber/Harvard Cancer Center institutional review board, and the study was conducted in accordance with the Declaration of Helsinki.

## Results

We completed 31 interviews across the four cases. Characteristics of the four case institutions are presented in Table 1. Despite the differing geographies and cancer program types, interview themes revealed more areas of similarity than difference across cases. Approximately 65% of the CFIR constructs or subconstructs had at least one area of thematic similarity across all four cases. A heat map showing the degree of similarity of themes within each construct and the cases in which each theme was represented are presented in Table 2. Below, we present key findings from the analysis organized by CFIR domain.

**Table 1:** Case characteristics

Characteristics	Institution A	Institution B	Institution C	Institution D
Cancer program classification	NCI designated comprehensive cancer center	NCI designated comprehensive cancer center	Comprehensive community cancer program	Comprehensive community cancer program
Geographic location (US)	Midwest	West	East	Midwest
Institutional EMR(s)	Epic	Epic	Athena, Cerner	Epic
Business model	Nonprofit	Nonprofit	Nonprofit	Nonprofit
No. participants	10	6	8	4
Roles represented	Administrator (3), Surgeon (4), IT & Informatics (2), Cancer Liaison Physician (1)	Administrator (2), Surgeon (3), Cancer Liaison Physician (1)	Administrator (4), Surgeon (2), IT & Informatics (1), Cancer Liaison Physician (1)	Administrator (1), Surgeon (1), IT & Informatics (1), Cancer Liaison Physician (1)

**Table 2:**
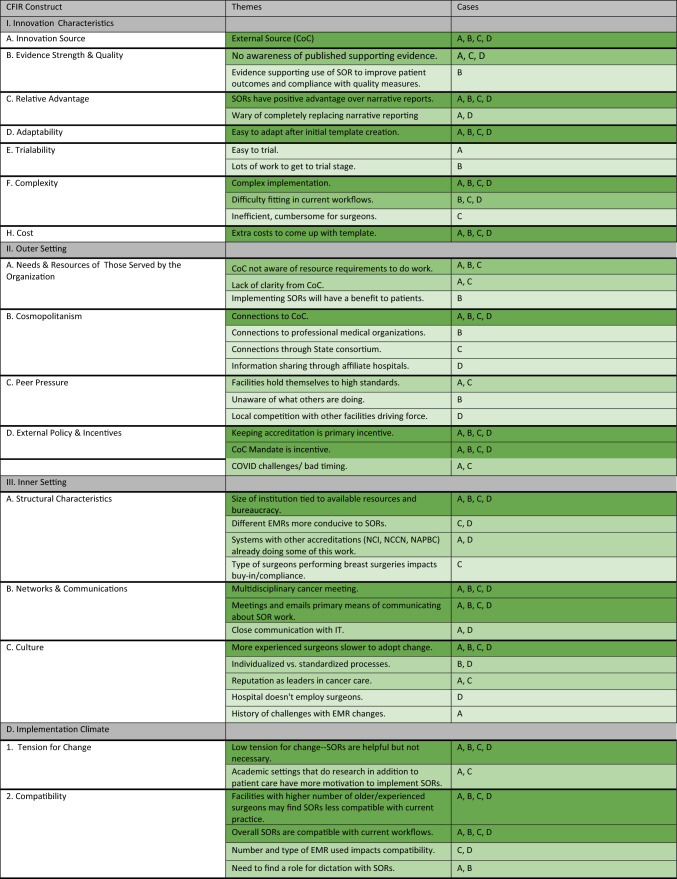

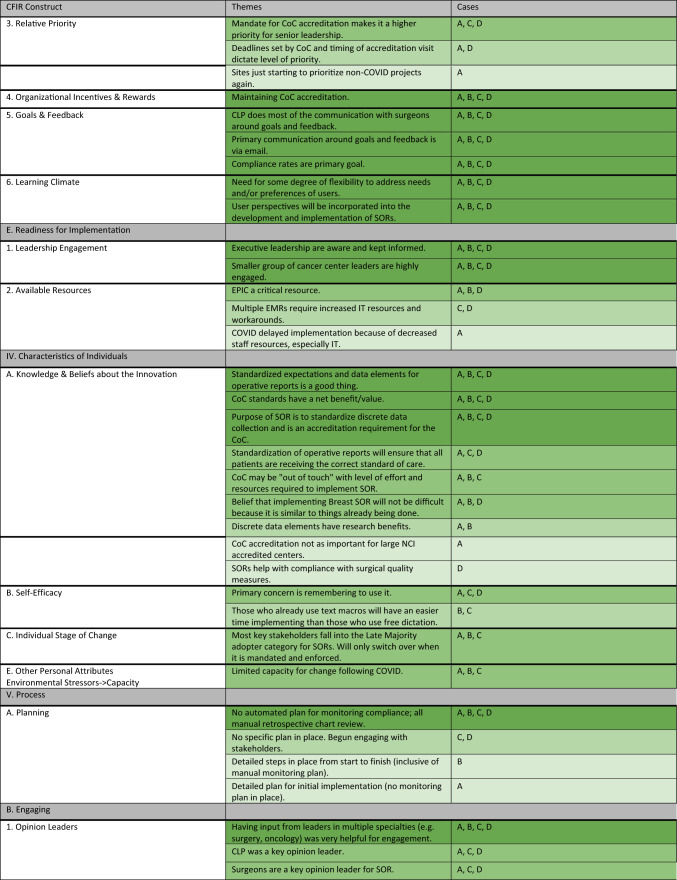

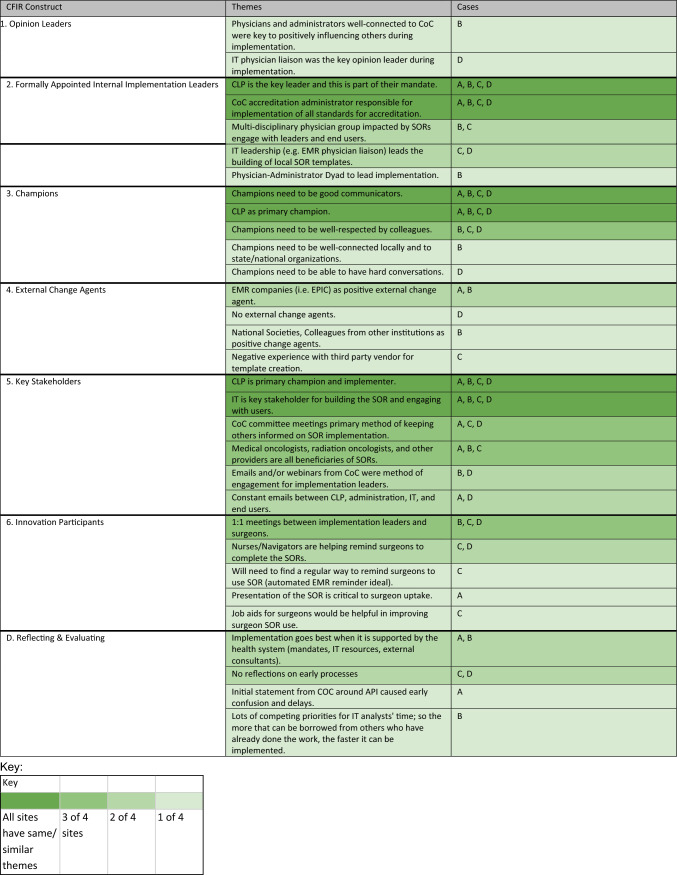
Similarity of themes across cases for CFIR constructs

### Innovation Characteristics

Interviewees from all cases reported understanding the advantages of SOR reporting and felt that there were benefits to using standardized checklists as part of documentation. Cases A and B cited benefits to capturing quality metrics and improving bioinformatics and EMR efficiencies. An interviewee from case B was the only person to mention being aware of evidence supporting the use of SORs to improve patient outcomes.

*“Yeah, [redacted] presented data from—there’s been some Canadian groups that have implemented it and they showed more compliance to guidelines and better patient outcomes.”*

Two cases (A and D) specifically noted that SORs should not replace narrative documentation for fear of missing important details not captured through standardized forms and should be added to existing narrative operative reports instead.

*“My bias would be to say that the narrative probably needs to be continued and protected just because it affords the opportunity to describe if things, like I said, were something unusual was encountered, and what the thoughts of the surgeons in the operating room was, and why you did what you did. I would imagine that that would be valuable from potentially a care perspective, but also a medical legal perspective.”*

Maintaining CoC accreditation was the main driver for designing and implementing the breast SOR in all cases. All cases also felt that implementing the SOR standards would be a complex undertaking due to challenges with surgeon buy-in, time and resource constraints, and difficulties incorporating it into workflows.

*"There's the technical challenge of being able to present this to our surgeons and what that looks like. And then part of that technical challenge is how do we incorporate that into their workflow in a way that it's something that they're already doing and not adding additional work to them."*

### Outer Setting

All four cases reported that maintaining CoC accreditation was the primary incentive for implementing SORs, although the level of importance of CoC accreditation varied across cases. Community cases C and D placed more value in maintaining CoC accreditation, because they felt that it was important for their reputation. One participant stated:

*“Oh, it’s very important to us. You know it makes a big deal, I think, to our population that we’re a CoC-accredited cancer center. We’re in a physician shortage area…so, to have something like this under our belt, it really means a lot to the population around us.”*

Case A, however, felt that CoC accreditation was less of a factor for patients who sought out care at large academic medical centers. One interviewee stated:

*“…People in smaller community programs that really need this accreditation for their marketing…are more likely to follow all the benchmarks, because they’re a small program and the CoC accreditation is really what helps distinguish them. Whereas programs, such as ours and large academic centers, the CoC accreditation helps but it’s not sort of the main driver that people come here for. They come here for the name.”*

Furthermore, interviewees from all cases expressed frustration with the implementation timeline and lack of explicit strategies provided by the CoC for how to implement SORs successfully, especially as they continued recovering from COVID challenges.

*“…when I said resources were limited, and we’re just now exiting a time where people had their full time job plus a COVID job…So, it was just a period of change where it was not a feasible time wise, or I would say financially not feasible at that time to use those extra resources during the midst of the heightened time of pandemic to roll out this.”*

When examining organizations’ relationships to external organizations and stakeholders, the four cases had very different levels of connection to the CoC beyond the cancer liaison physician (CLP), as well as other state and national medical organizations. For example, case A had multiple clinicians with close connections to the CoC and was involved in providing feedback on potential CoC standards. One interviewee explained:

*“…we got together people from ACOSOG and CALGB and then from SWOG and SSO and ASBRS and for breast we came up with guidelines. I actually wrote the lumpectomy chapter, but I edited the whole thing.”*

Case B had several members of various cancer committees who had formal and informal contacts at the CoC and in several other medical organizations who helped improve their level of awareness around SORs. Cases C and D reported being connected to the CoC through one individual (the CLP and an external consultant) and received updates on standards via mass communication emails from the CoC or at conferences.

### Inner Setting

All cases noted that IT resource availability was a limiting factor in the timeliness of implementation. Those cases (A, B, D) who used Epic as their EMR all referenced it, and Epic trainers and other personnel, as key resources in the development and implementation of their Breast SORs. One interviewee stated:

*“So initially, looking at this when we asked the Commission on Cancer about this, they were like, Well, you’re kind of on your own. It’s up to your individual IT Epic application builders to help you figure out how to make this work. So that was a problem. They also mentioned having some kind of Epic integration or API, and they never, never and to this day have never given any overview of what that is or how it would work or how it would be incorporated into workflow. They did address one of the concerns, at least for organizations that use Epic, is that they have partnered with Epic to be able to have it as part of what is known as Foundation Build.”*

Cases C and D had multiple EMRs, resulting in a need for increased IT resources and workarounds to develop the SOR. An interviewee commented:

*“The challenge is that when you have systems that not—don’t communicate with each other and the geographically, you don’t have physicians that are in the same building or see each other that often, it creates a challenge. Thankfully, most of the surgeons are in Athena, which is a one centralized system. But the challenge is then when they go into the hospital to do all the surgery and everything, they’re going to be working in Cerner… So, and then the other challenge is they’re not interfaced. So, they’re—it’s not like we’re able to freely communicate within the system just by using interfaces. So that’s something we need to work on.”*

All cases noted that the creation of the SOR template required considerable time, with cases B and C specifically noting that having a standardized example template would have saved hospitals time in the development and implementation of their Breast SORs.

*“I imagine by creating those discrete fields, they need to be connected in Epic. And it looks like even further down in this document, it talks about the integration could include HL seven, which I don’t really understand the need for that, but it sounds pretty extensive, not just creating a table. So I think we need to dig into that a little bit more.”*

*“…my initial perception was that there would be more templated analyst build already developed that we could sort of copy or adopt or whatever you may do. But I was hoping there could—we could leverage more of the work that’s being done in these pilot institutions. And perhaps that’s the ultimate goal. But right now, if we were to try to do it ourselves, I think the time and the total FTEs that we have to cover all of our priorities is really the limiting factor.”*

All four cases reported a low tension for change to SOR, stating that they were helpful but not necessary. One interviewee stated:

*“I don’t know if putting it in their operative report is going to make them change their operation to meet those standards. So, I’m not sure if it’s one hundred percent needed.”*

There was also the impression that the concept for SORs arose to benefit mostly for research purposes, which did not resonate with practitioners in community institutions, keeping tension for change low.

*“…The whole effort of the critical elements was initiated … with the complaint that she couldn’t do meta-analysis because the surgical procedures were all done differently. And so, OK, that I understand that’s important, but it didn’t register with me, one of the all-time important aspects of how a clinical practice needed to change in the community.”*

#### Characteristics of the Individual

All four cases generally had a positive view of the CoC and felt that the accreditation standards developed by the CoC had a net benefit to cancer care. There were mixed opinions on the benefits that breast SORs, however. Some interviewees felt that standardizing expectations and data elements for operative reports was valuable and had both research and patient care benefits. One participant stated:

*“…I think if you have standards and if you follow procedure and if you gather your metrics, that’s how you can review the data and see if we’re best serving our patients, if there’s other changes we need to make where we could be providing the best quality of care for our patients. So, I think that’s what having accreditation do[es]. They hold you responsible and make sure that you’re living up to care that the patients all deserve.”*

Others, however, did not feel that the SOR would lead to a change in practice. Interviewees commented:

*“I feel like for people that are in practice who have been in practice for a long time, though, I don’t think it’s necessarily going to change the way they practice. So, for maybe for them, it’s not as beneficial. And again, hopefully they’re meeting those technical requirements anyway. But I would say for research and for training purposes, it would be helpful.”*

The academic medical center cases (A and B) also noted the research benefits of the discrete data elements provided by SORs, but the community cases (C and D) focused on compliance with quality measures and improving quality of care. One participant from case B noted:

*“…we’re very interested in moving in that direction because it’s what lays the groundwork for capturing discrete data that otherwise would require manual chart review, thousands of person hours of labor and so on. Whereas if you can do this in an elegant fashion and capture the data anyway, then you are able to use it for quality improvement work, population health purposes and research.”*

While a participant from case D discussed the purpose and benefit of SORs as:

*“…my understanding is they use it to pull out in a standardized fashion so they can interrogate the medical record to find out compliance with the quality measures associated with the surgeries.”*

Interviewees from three cases (A, B, C) felt that the SOR implementation timeline provided by the CoC indicated that they may have underestimated the time and resources that it would take to develop and implement the Breast SORs, with it being a higher burden for community institutions.

*“And this might be where the CoC may be overstepped a little bit to assume that everyone has the resources to implement this level. You know, we can make it happen if there is a true benefit, and but six to nine months of analyst time is pretty significant.”*

*“I believe that it’s much easier for bigger institutions to implement this…because of the resources that we have, for example,…we have IT support resources that can be dedicated just for that implementation of the operative synoptic reporting. I think in a smaller community hospital, it really is dependent on how much support the administrative people gives to the cancer registry, because this type of standard implementation cannot be achieved by just one person. It has to be a combination of the liaison physician, someone from IT, someone from administration that will push it to all the departments that needs, you know, that is needed in this implementation, as well as the participation of the surgeons the surgeons are the biggest, you know, without their participation and support and, you know, able and willing to do this.”*

#### Process

At the time of the interviews, each of the four cases had completed very different levels of planning for the implementation of SORs. Case A had specific steps in place for initial implementation, including building the template and engaging with different stakeholders, but had not developed a clear plan for how to monitor compliance with the standard. Case B, however, had created a detailed plan that did encompass how to monitor for compliance with the reporting standard. Cases C and D had very little specific steps planned for their implementation processes, but both had begun to engage with key stakeholders. Similarly, the degree to which key stakeholders were involved in the development and implementation of the SORs varied across the cases. For example:

*“… I probably haven’t involved all of the stakeholders who should be. The individual who’s in charge of quality measures, quality improvement, probably needs to be more involved in this. The director of the cancer program needs to be at least aware that this is a small change, but it’s a change that is a CoC standard. And I should think more about who are other stakeholders that, even though they don’t dictate operative reports, need to be aware that this is a change that is a requirement.”*

*“We would then get our analysts lined up and we would have a work group set up that would include probably our lead physicians that have been involved to take. So, Dr. X, Dr. Y, Dr. Z, to be somewhat multidisciplinary, multispecialty, and then we would draft out the synoptic op reports and then circulate those to the other surgeons on those teams. So really, that’s part of kind of more the change management, stakeholder engagement, just making sure that everyone understands there’s a requirement coming, they feel like part of the process and like they’ve provided feedback in the process and then, and then once we get sign off, which may be a process in itself, would kind of wait on the EPIC build.”*

Finally, there was significant overlap across the cases in both who was critical to involve in the implementation process and how to engage with them effectively. The CLPs, who are the designated physician quality leaders of the hospital’s cancer committee, played a critical role in leading the implementation of SORs in all four cases; this was considered part of their mandate in taking this position. They were the primary person engaging with key stakeholders across all levels of the organization and were also seen as a key opinion leader for SORs, regardless of their specialty. One participant stated:

*“Most of the cancer liaison physicians are medical oncologists or surgeons. So, I don’t think there is a specific skill that’s needed. It helped that [redacted] is a surgical oncologist. So, because it has to do with the operative report, [they’re] more familiar with it. But I think anyone could, you know, any clinician would have been able to implement this or, you know, champion in implementing this.”*

## Discussion

Adoption of previous CoC standards has varied widely across accredited sites, limiting their ability to positively impact the quality and safety of cancer care. Previous standards have come with limited implementation resources and support from the CoC.^26–28^ From the perspective of the accredited sites, the CoC’s educational resources aimed at surgeons were not adequate implementation support for the new breast SOR standards. Our four-site case comparison of two large academic centers and two community hospitals revealed that there were more areas of similarity than difference found within the context and implementation approaches. The similarities we identified highlight that barriers to implementation cannot be overcome with solely the educational resources provided by the CoC. There were also several key themes that appeared in multiple CFIR domains, such as limitations in IT resources, an acknowledgement of the advantages of CoC accreditation, SORs, and standardized documentation, and the persistent low tension for change. Several key stakeholders, including the CLP, IT analysts and administrators, and surgeon end-users also appeared frequently in the themes across multiple CFIR domains, highlighting their importance to successful implementation.

Across cases, interviewees expressed both low tension for change and recognition of benefits from standardized documentation through SORs; two themes that initially appear contradictory. This is most likely explained by the complexity of implementing SORs and a lack of perceived universal benefit for surgeons and patients. With low tension for change, interviewees questioned whether improved documentation would alter clinical practice or impact patient outcomes, particularly when the implementation required significant effort. While some saw value in standardized documentation, especially for academic sites interested in research, this benefit was less motivating for physicians in community hospitals. Furthermore, only one case could identify clear evidence linking SORs to better patient outcomes, making the justification for adopting a complex intervention less compelling when resources are limited.

Our findings highlight both the importance of accrediting bodies creating standards for achieving large-scale quality improvement and an opportunity to work with health facilities to provide more implementation support for their standards. Studies have found mixed results on the impact of hospital accreditation on patient outcomes.^17,29–31^ Given that the standards are uniform for all hospitals with a given accreditation, some of the variable impact on patients may be due to how successfully these standards are implemented. In this study, all cases saw the value of the accreditation standard for SORs but expressed frustration that the CoC provided limited guidance on implementing them within the timeline provided, despite their competing priorities and concerns.

The CoC’s accreditation standards in breast surgery emphasizing synoptic documentation of operative techniques is unique from its prior standards in two distinct ways: requirement of specific documentation by surgeons in the electronic medical record and the need for IT support in implementation of the documentation template. Thus, our findings support the need for tailored implementation support that is not only surgeon-focused but also informatics specific. The CoC’s initial implementation effort focused on a series of educational resources (i.e., webinars) to inform surgeons about the new SOR standards.^10^ Based on the data presented here, cases had similar levels of knowledge and beliefs around SORs, but tension for change remained low for all of them. Other common challenges (e.g., cost, complexity, capacity for change) remained unaddressed with existing CoC implementation resources. For example, several interviewees mentioned that having an SOR template from the CoC for their IT departments would have made implementation significantly easier. Interviewees also mentioned costs associated with developing their own SOR template and linking the templates with the appropriate operative cases, which had a more significant impact on community hospitals compared to the larger academic institutions.

There are examples of CoC committees taking a more active role in facilitating implementation of best practices in cancer care. In 2022, the CoC sponsored the Just ASK Quality Improvement project and study to better integrate smoking assessments into routine cancer care. Approximately half (49%) of CoC-accredited institutions participated in this quality improvement project, which earned them credit towards CoC accreditation standards. A 12-month follow-up on participating institutions revealed significant improvements in the evidence-based Ask, Advise, and Assist practices.^32^ This study demonstrates that when accrediting bodies, such as the CoC, become implementing partners for evidence-based practice, they can achieve large-scale impact on quality of care and patient outcomes. More consistent alignment and collaboration between the committees within accrediting organizations (e.g., standards, accreditation, and quality improvement committees) has the potential to achieve improved implementation outcomes and more consistent positive impacts associated with hospital accreditation. The findings of this study can be informative in designing intervention studies. We have previously demonstrated implementation strategies that are matched to specific barriers.^33^ For example, the CLP, regardless of their specialty, was frequently identified as the main person who was responsible for assessing the implementation needs of the new accreditation standards and communicating those needs to other stakeholders, including IT. Thus, the CoC could provide CLPs with more explicit instructions on how the CLPs can assess their local needs and communicate that effectively with IT.

While there were many similarities between the four cases, there were important differences identified through this analysis. For example, cases had different levels of connection to the CoC and other professional organizations, which impacted when and how they received information and updates related to the SOR standards. This likely impacted the wide variation in progress made on implementation planning seen across the cases. The case’s culture, social networks, and resources resulted in differences in levels of stakeholder engagement, varying timelines for implementation, and diverse support needs to facilitate use of the SORs.^34^ It has been established that context is an important mediator in the impact of barriers and facilitators on the implementation of improvement initiatives.^35^ While it is impractical to assume that accrediting bodies, as more active partners in implementation, could address all barriers, additional research to understand the barriers common to different contextual factors would allow accrediting bodies to better identify and refine the implementation strategies most likely to be effective for different scenarios.

There are limitations to this study. First, because all cases were CoC-accredited and the SOR was mandated to maintain accreditation, there is a risk of social desirability bias in interviewees’ responses. Second, although we included both academic and community institutions in this case comparison, there are limitations to the generalizability of our conclusions inherent in qualitative research and the limited number of sites included. Third, the interviews were conducted during the COVID-19 pandemic, which may have influenced some of the participant’s perspectives. However, many of the themes identified in our study were not directly related to the pandemic, which is why we believe the findings of this study are still relevant. Furthermore, there may be fundamental differences in the cases that agreed to participate in this analysis and others who were not included, which may bias the results. Finally, themes were attributed to cases where at least one interviewee discussed concepts that were encompassed by that theme. The variation in the number and characteristics of interviewees across cases may have impacted the final analyses and conclusions drawn. For example, the lack of specific mention of a theme at a case does not necessarily mean that this theme was not applicable to that case, but may reflect the number and type of interviewees at a site.

## Conclusions

This CFIR-guided case comparison of four CoC-accredited institutions revealed key areas of similarity and difference in context, SOR knowledge and attitudes, and implementation approaches that are critical to better understanding specific challenges associated with the implementation of Breast SORs. This information can be used by the CoC to test how development of additional implementation support and resources that addresses common challenges identified here impacts adoption of the SOR standards. Moving forward, the CoC and other accrediting bodies should consider more collaboration and alignment between their standards, accreditation, and quality improvement arms to become a more active implementation partner for evidence-based practice and realize more consistent positive impacts associated with hospital accreditation.

## Supplementary Information

Below is the link to the electronic supplementary material.

Supplementary file1 (DOCX 25 kb)

## Data Availability

Data are available upon reasonable request.
