# Supplementary material for: Bridging Operative Standards to Clinical Practice: A Case Comparison of Synoptic Operative Report Implementation in Breast Cancer Surgery
Source: Ann Surg Oncol. 2026 Jan 27;33(5):4482–93. doi: 10.1245/s10434-025-19042-6 (PMC13083452; doi:10.1245/s10434-025-19042-6)
Supplement: Supplementary file 1 — Supplementary file1 (DOCX 25 kb) [file 10434_2025_19042_MOESM1_ESM.docx]

**SEMISTRUCTURED INTERVIEW GUIDE**

**Introduction**

Is this still a good time for you? Are you in a place where you can be free from distractions and feel free to give candid responses?

[Indicate if anyone else is on the call (eg, for training purposes); make sure that this is OK.]

Thank you for agreeing to participate in this interview. The aim of the study is to help us understand more about the implementation of the CoC Operative standards in breast surgery and the synoptic operative report (SOR; abbreviated this way only for text; please call them synoptic operative report during the interview).

Our discussion should last between 30 and 45 minutes. Your responses will help us understand how best to implement SOR in practice. All of your responses will remain confidential. You may choose to stop the interview at any time, and there is no penalty to your or your organization for not completing the interview. If you complete the interview, we will offer you a Amazon.com gift card to thank you for your time.

Before we begin, we would like to ask your permission to audio record our discussion (for research and training purposes). Would it be OK with you if I record this call? The interview will be transcribed, however, your name or any personal identifiers will not be associated with any of the notes. The audio recordings will be deleted once the project is complete. If you have any questions, I can provide contact information for the PI. [Ask other person on call to begin recording.]

Do you have any questions before we begin?

To start, can you tell us if you have heard about the CoC’s new accreditation standard synoptic operative report (SOR)?

(Prompt: If so what you know about the CoC’s SOR?)

(If participant does not know, then explain CoC’s SOR.)

SORs are template checklist format op report that documents certain technical aspects of the surgery. For the new COC accreditation standard, the breast SOR elements highlight technical aspects of the sentinel lymph node biopsy and axillary lymph node dissection. When we refer to “SOR implementation” throughout the interview, we mean the surgeon using an electronic templated checklist to document the technical aspects predefined by the CoC with regards to sentinel lymph node biopsy and axillary lymph node dissection (either in place of a narrative operative report or in conjunction with.

[Instruction to interviewer: depending on stakeholder role (surgeon, administrator, IT specialist) use the designated interview guide]

**Stakeholder: Surgeon**

| Question | CFIR domain/ TDF |
| --- | --- |
| To start, please share with me your thoughts and impression about using Synoptic Operative Reports in clinical practice. |  |
| How did you first hear about SOR? | Inner Setting: Networks & Communications |
| Please talk about your willingness to use SOR.  (Prompts: Any competing tasks that make it difficult?) | Individual Characteristics: motivation and goals (TDF) |
| How do people in your institution perceive CoC and SOR? | Inner Setting: Culture |
| Can you tell me your impression about the general receptivity in your organization to implementing the SOR? | Inner Setting: Implementation climate |
| How does professional networking that you engaging in influence your perspective of SOR?  (Prompts: Local or national conferences? Social media?) | Outer Setting: Cosmopolitanism |
| To what extent do you think your colleagues are implementing the SOR? Does this influence your decision to use SOR?  (Prompts: own institution colleagues or colleagues nationally) | Outer Setting: Peer Pressure |
| Could you please talk about the extent to which you feel that you need to use SOR?  (Prompts: How much?) | Individual Characteristics: Social influence (TDF), Individual identification with the organization |
| What kind of supporting evidence about the effectiveness of SOR have you heard? | Intervention Characteristics: Evidence Strength & Quality |
| What do you think are advantages of using SOR?  (Prompts: benefits?)  (Prompts: If so, what are the benefits? To you, patients, oncology providers, primary care providers, the cancer program, the health care system … in terms of what it will allow you/them to do, feel, act, and think … long term and short term?) | Intervention Characteristics: Relative advantage |
|  | Individual Characteristic: Beliefs about consequences (TDF) |
| What do you think are disadvantages of using SOR?  (Prompts: any potential downsides?)  (Prompts: If so, what are they? To you, patients, oncology providers, primary care providers, the cancer program, the health care system … in terms of what it will allow you/them to do, feel, act, and think … long term and short term?) | Intervention Characteristics: Relative advantage |
| From your perspective, do the advantages of SOR use outweigh the disadvantages? Why? | Individual Characteristic: Beliefs about consequences (TDF) |
| Please describe any incentives offered to you to use SOR.  (Prompts: If so, what kind[s]?) | Individual characteristics: motivation and goals (TDF) |
| The SOR is a CoC mandate. Does this influence your thoughts about SOR?  (IF SO, how)  (IF NOT, then why) | Outer Setting: External policies |
| Have you used SOR in clinical practice, either at your current institution or prior ones? | Intervention Characteristics |
| IF YES: Can you elaborate on the circumstance? When have you used it? |  |
| Do you think SOR can be adapted in your setting? | Intervention Characteristics: Adaptability |
| (Prompts: any perceived barriers or facilitators?)  IF YES: Why?  IF NO: Why not? |  |
| What kinds of resources are available to you for using SOR. If none, which would be helpful?  (Prompts: If so, what kind[s]? If not, what kind[s] would be helpful? Financial, computer access near OR, electronic medical record template, training, and human resources, leadership?) | Individual Characteristics: Environmental context and resources (TDF) |
| I’d like to ask you what you do with respect to implementing SOR. Walk me through the steps you take to implement SOR.  Can you describe the plan for implementing SOR?  Who else is involved in the process? | Process |
| Did someone ask you to implement SOR? | Process: Planning |
| Who needs to be involved to implement SOR in your institution?  (Prompts: electronic medical record changes?) | Inner Setting: Structural characteristics |
| Tell me about how EPIC [interview modifies to programs EMR system] impacts your ability to implement SOR? | Inner Setting |
| Did you develop the electronic template for SOR? | Process: Planning |
| Would you please discuss how your role in implementing SOR came about?  Are you able to/have you contributed to decisions about how SOR are used in your organization?  Was the task delegated to you? Or you volunteered? | Process: Engaging |
| Who is championing this?  How was this person chosen to lead this? What experiences or skills do they have to qualify them for the role?  What additional skills/experiences do you think should be added to the team to complement this leader? | Process: Formally appointed internal implementation leaders |
| Other than formal implementation leaders, are there people in your organization who are likely to champion the intervention?  What kind of actions do you think this individual will exhibit? (Prompts: For example, helping get senior leaders on board, helping solve problems? Or a small role?) | Process: Champions |

**Stakeholder: Administrator**

| Question | CFIR domain/ TDF |
| --- | --- |
| To start, please share with me your thoughts and impression about using Synoptic Operative Reports in clinical practice. |  |
| How did you first hear about SOR? | Inner Setting: Networks & Communications |
| How do people in your institution perceive CoC and SOR? | Inner Setting: Culture |
| Can you tell me your impression about the general receptivity in your organization to implementing the SOR? | Inner Setting: Implementation climate |
| How does professional networking that you engaging in influence your perspective of SOR?  (Prompts: Local or national conferences? Social media?) | Outer Setting: Cosmopolitanism |
| To what extent do you think your colleagues are implementing the SOR? Does this influence your decision to implement SOR?  (Prompts: own institution colleagues or colleagues nationally) | Outer Setting: Peer Pressure |
| Could you please talk about the extent to which you feel that surgeons need to use SOR?  (Prompts: How much?) | Individual Characteristics: Social influence (TDF), Individual identification with the organization |
| What kind of supporting evidence about the effectiveness of SOR have you heard? | Intervention Characteristics: Evidence Strength & Quality |
| What do you think are advantages of using SOR?  (Prompts: benefits?)  (Prompts: If so, what are the benefits? To you, patients, oncology providers, primary care providers, the cancer program, the health care system … in terms of what it will allow you/them to do, feel, act, and think … long term and short term?) | Intervention Characteristics: Relative advantage |
|  | Individual Characteristic: Beliefs about consequences (TDF) |
| What do you think are disadvantages of using SOR?  (Prompts: any potential downsides?)  (Prompts: If so, what are they? To you, patients, oncology providers, primary care providers, the cancer program, the health care system … in terms of what it will allow you/them to do, feel, act, and think … long term and short term?) | Intervention Characteristics: Relative advantage |
| From your perspective, do the advantages of SOR use outweigh the disadvantages? Why? | Individual Characteristic: Beliefs about consequences (TDF) |
| The SOR is a CoC mandate. Does this influence your thoughts about SOR?  (IF SO, how)  (IF NOT, then why) | Outer Setting: External policies |
| Have you seen SOR in clinical practice, either at your current institution or prior ones? | Intervention Characteristics |
| IF YES: Can you elaborate on the circumstance? When have you used it? |  |
| Do you think SOR can be adapted in your setting? | Intervention Characteristics: Adaptability |
| (Prompts: any perceived barriers or facilitators?)  IF YES: Why?  IF NO: Why not? |  |
| What kinds of resources are available to surgeons to use SOR. If none, which would be helpful?  (Prompts: If so, what kind[s]? If not, what kind[s] would be helpful? Financial, computer access near OR, electronic medical record template, training, and human resources, leadership?) | Individual Characteristics: Environmental context and resources (TDF) |
| I’d like to ask you what you do with respect to implementing SOR. Walk me through the steps at your institution to implement SOR.  Can you describe the plan for implementing SOR?  Who else is involved in the process? | Process |
| Did someone ask you to implement SOR? | Process: Planning |
| Who needs to be involved to implement SOR in your institution?  (Prompts: electronic medical record changes?) | Inner Setting: Structural characteristics |
| Tell me about how EPIC [interview modifies to programs EMR system] impacts your ability to implement SOR? | Inner Setting |
| Would you please discuss how your role in implementing SOR came about?  Are you able to/have you contributed to decisions about how SOR are used in your organization? | Process: Engaging |
| Who is championing this?  How was this person chosen to lead this? What experiences or skills do they have to qualify them for the role?  What additional skills/experiences do you think should be added to the team to complement this leader? | Process: Formally appointed internal implementation leaders |
| Other than formal implementation leaders, are there people in your organization who are likely to champion the intervention?  What kind of actions do you think this individual will exhibit? (Prompts: For example, helping get senior leaders on board, helping solve problems? Or a small role?) | Process: Champions |

**Stakeholder: EMR engineer**

| Question | CFIR domain/ TDF |
| --- | --- |
| How did you first hear about SOR? | Inner Setting: Networks & Communications |
| How do people in your institution perceive CoC and SOR? | Inner Setting: Culture |
| Can you tell me your impression about the general receptivity in your organization to implementing the SOR? | Inner Setting: Implementation climate |
| The SOR is a CoC mandate. Does this influence your thoughts about SOR?  (IF SO, how)  (IF NOT, then why) | Outer Setting: External policies |
| Have you seen SOR in clinical practice, either at your current institution or prior ones? | Intervention Characteristics |
| IF YES: Can you elaborate on the circumstance? When have you used it? |  |
| Do you think SOR can be adapted in your setting? | Intervention Characteristics: Adaptability |
| (Prompts: any perceived barriers or facilitators?)  IF YES: Why?  IF NO: Why not? |  |
| What kinds of resources are available to surgeons to use SOR. If none, which would be helpful?  (Prompts: If so, what kind[s]? If not, what kind[s] would be helpful? Financial, computer access near OR, electronic medical record template, training, and human resources, leadership?) | Individual Characteristics: Environmental context and resources (TDF) |
| I’d like to ask you what you do with respect to implementing SOR. Walk me through the steps at your institution to implement SOR.  Can you describe the plan for implementing SOR?  Who else is involved in the process? | Process |
| Did someone ask you to implement SOR? | Process: Planning |
| Who needs to be involved to implement SOR in your institution?  (Prompts: electronic medical record changes?) | Inner Setting: Structural characteristics |
| Tell me about how EPIC [interview modifies to programs EMR system] impacts your ability to implement SOR? | Inner Setting |
| Would you please discuss how your role in implementing SOR came about?  Are you able to/have you contributed to decisions about how SOR are used in your organization? | Process: Engaging |
| Who is championing this?  How was this person chosen to lead this? What experiences or skills do they have to qualify them for the role?  What additional skills/experiences do you think should be added to the team to complement this leader? | Process: Formally appointed internal implementation leaders |
| Other than formal implementation leaders, are there people in your organization who are likely to champion the intervention?  What kind of actions do you think this individual will exhibit? (Prompts: For example, helping get senior leaders on board, helping solve problems? Or a small role?) | Process: Champions |

Those are all of the questions that I had planned for today. Is there anything that we have not covered during the interview that you feel is important for me to understand the implementation of SOR?

**Almost done. We just have a few quick background/demographic questions we would like to ask you.**

1. In what year were you born/How old are you? What is your race/ethnicity?
2. How many years have you been in your current organization? What is your main role at the institution (if surgeon, then what is breakdown of practice in breast patients)?
3. {If surgeon} Are you board certified? Did you complete a fellowship(s)? In which field? Do you work full or part time? How many years have you been in practice?
4. Is this the only hospital that you operate in or are there other hospitals?

Finally, I am wondering whether you are willing to provide contact information for any other providers in your organization who will need to use SOR or those who may be involved in the implementation process.

[Stop recording.]

Thank you for your time.
